# Clinical Lecture on Diphtheria

**Published:** 1865-10

**Authors:** Edw. Headlam Greenhow

**Affiliations:** Assistant Physician to the Middlesex Hospital


### CLINICAL LECTURE ON DIPHTHERIA.

By EDW. HEADLAM GREENHOW, M. D.,
*Assistant Physician to the Middlesex Hospital.*

GENTLEMEN:

I propose to bring before you the subject of Diphtheria, and to take as the basis of my lecture two cases which have recently been in the hospital, and which were characteristic examples of the two principal varieties of this formidable complaint—namely, of that form in which the urgency of the case is due to the local manifestations of the disease, and of that other form in which the danger arises from the general constitutional affection. The former of these is especially characterized by the existence of symptoms of apnœa, and the pressing danger is caused by the more or less complete occlusion of the air passages by the membraniform exudation from which the disease derives its name of diphtheria. The latter, on the other hand, is characterized by the predominance of symptoms of asthenia caused by the intensity of the general disease, and the danger to be apprehended is the general exhaustion of the vital powers. You should, however, fully understand, that although these two forms of diphtheria are so diverse in their more silent characters, and the kinds of danger which attend them, there is yet no doubt of their being, as I have said, only varieties of one and the same disease, for they not only occur constantly during the same epidemic, but very often also in the same household at or about the same time. I have seen many examples of this, and one, which occurred only a few months ago, was especially striking, on account of the severity of both forms, causing death rapidly in both cases. I was called to see a young gentleman aged fifteen, who had come home from school, I believe, with the complaint, and was suffering from most urgent laryngeal symptoms of which, in fact, he died the same evening, almost immediately after the operation of tracheotomy. Four days afterwards his sister, aged eight years, was taken ill with diphtheria of the other, the asthenic form, which also ran an unusually rapid course, and proved fatal on the fifth day of her illness, without the supervention of any laryngeal symptoms. Another proof of the identity of the disease in the two different forms is, that although, in many cases, their separate characters are as sharply marked as in my description above, yet other cases occur side by side with them which partake more or less of the characterr of both forms. I say more or less, because in fact one of the two classes of symptoms does usually predominate.

Although the two varieties of diphtheria to which I propose directing your attention to-day are the most important, I must remind you that they are by no means the only forms of this disease. In every epidemic there are many cases in which neither are the air-passages involved nor are there any urgent symptoms of general constitutional affection. Many of these would perhaps at another time be regarded merely as cases of common inflammatory sore-throat; but, occurring as they do at the same time, and frequently in the same household with characteristic cases of diphtheria, we cannot but refer them to the same category. Several of you saw, the autumn before last, a rather severe case of diphtheria, which came from a house in the vicinity of the hospital in which sore-throat had previously been prevalent. The lad, aged sixteen, was a shoemaker's apprentice, and slept in the same room with three other boys. The family consisted of eight or nine persons, five of whom, including the three fellow-apprentices, had been under my care as out-patients in quick succession during the preceding fortnight, for sore-throat of varying intensity, but unaccompanied by exudation. Lastly, but still forming a part of the same epidemic—if I may be allowed to apply the term to so limited an outbreak of disease—this lad presented himself, with symptoms of so great prostration that we were compelled to take him into the hospital. His fauces were coated on both sides with the characteristic false membrane, and although he made a good recovery, his illness was severe. Albumen was found in his urine on the day after his admission, and he only regained health and strength after a prolonged and tedious convalescence. Again, cases characterized it is true by more or less diphtheritic exudation in the fauces, but unattended by any urgent symptoms, form a large proportion of every epidemic. Sometimes it even happens that almost all the cases in particular epidemics are of this mild kind. Such cases usually recover under any rational treatment, and it is the consequent great apparent success in treating them which has sometimes led even honest and worthy practitioners to promulgate as a specific for diphtheria some medicine with which they have treated large numbers of cases; the truth being that by far the greater proportion of these cases required only common care, and would probably have recovered without any medical treatment at all. But although these mild cases so often do well with any or no particular medicine, I must not dismiss them without a word of caution to you, on the one hand, against over-treating them, and, on the other, against neglecting them. I have seen serious mischief ensue from what I must term meddlesome treatment of such cases, especially when in the form of local applications; and yet even the mildest case requires careful watching, because, either by the invasion of the air-passages or by the accession of constitutional symptoms, a case which in i s first stages appeared of the mildest kind may subsequently assume a most serious form.

With these preliminary observations, and begging you to bear in mind that I can only bring a small section of my subject, so to speak, before you to-day, I proceed to consider the first of the two grave forms of diphtheria which I have described, as it was exemplified in the more recent of our two hospital cases.

Mary Ann M——, aged eleven years, was admitted into Northumberland ward on the 24th of April, under the care of my colleague, Dr. Thompson. She had been ailing with cold for about a week, and had had sore throat from the first. Two days before admission she had commenced coughing, and at the same time her voice had become hoarse; but, notwithstanding her indisposition, she had continued able to play about with her companions as usual in the open air until the afternoon of Sunday, April 23d, when dyspnœa came on more decidedly, the cough and hoarseness increased, and she became so ill that on the following morning her mother procured her admission into the hospital. At the time of admission her breathing was difficult, labored, and stridulous, and she spoke in a faint, husky voice. Her face was flushed, and the expression of her countenance anxious. Her skin was hot, pulse about 140, respirations about 24 in a minute. Mr. Waymouth, the clinical assistant, under whose observation she first came, states that at the time of her admission there was a small patch of false membrane on the fauces; but when I saw her, two or three hours later, this had disappeared. The case seemed so urgent that Dr. Thompson requested his colleagues, including myself, to see the patient, for the purpose of considering the propriety of performing the operation of tracheotomy. I found both tonsils enlarged, somewhat ragged-looking and reddened; the pillars of the fauces were also of a dusky-red color, and the glands at the angles of the jaw, especially those on the right side, were slightly enlarged. The tongue was moist, and coated with a white fur. The urine was normal in appearance, and contained no albumen. On examining the anterior part of the chest, I found a deficiency of resonance in the left infra-clavicular region, and the respiration on both sides of the chest sibilant, with slight rhonchus in the upper part of the left lung. I was informed that there was dulness on percussion over the greater part of the left side of the thorax posteriorly, but the child was so ill that I did not attempt to verify this fact for myself. Immediately after the consultation tracheotomy was performed by Mr. Moore, and I shall presently state to you what were my views of the case, and the grounds on which I advocated a decision in favor of operating. From two and a-half to three ounces of blood were lost during the operation, and the pulse at once fell to 128, but shortly became exceedingly feeble. The respirations became comparatively tranquil, and the child fell into a quiet sleep. At eleven P. M., the pulse was 140, the respirations 34; the patient had slept well at intervals, and had partaken very freely of the strong beef-tea and brandy ordered for her. On the 25th it was reported that she had passed a good night and had taken abundance of nourishment. She was perfectly calm, her pulse from 130 to 140; but the respirations, though unembarrassed, were very frequent, being nearly 50 in a minute. The breathing was found to be tubular, and the percussion resonance over the whole of the thorax posteriorly; the respiration over the right scapula was also slightly bronchial; the deficiency of resonance and bronchial breathing below the left clavicle remained as before the operation. Throughout the day and night she continued stationary, coughing a good deal, but expectorating freely, until early on the morning of the 28th, when she became restless and began to have difficulty in raising the expectoration. During the day her breathing became more and more embarrassed, and gradually sank, and died at half-past four P. M., about 48 hours after the operation.

At the post-mortem examination a shallow, ragged ulcer was found on the surface of each tonsil, but both throat and fauces were free from exudation. A small patch of false membrane was lying loose on the under surface of the epiglottis, and the larynx and also the trachea for the space of an inch and three-quarters downwards, were almost entirely lined with a tough false membrane of about the thickness of kid leather, for the most part lying loosely on the mucous surface, but here and there so firmly attached as to require much force to tear it away. The incision made by the operation had passed directly through this membrane. The larynx, trachea and bronchi contained a large quantity of thick, tenacious, muco purulent secretion. The mucous membrane of the larynx and trachea was generally somewhat reddened, and presented distinct patches of a still deeper redness. On tracing the left bronchus downwards from its origin, the smaller tubes were found to be inflamed and filled with thick mucus. The upper lobe of the left lung was collapsed, and the posterior part of the lower lobe was dark colored and much congested. The right lung was also slightly congested, but otherwise appeared to be normal. The rest of the viscera were healthy.

I have spoken of this case as illustrating one of the forms of diphtheria, because I regard it as having really been a case of that disease, and not one of ordinary croup; and I am led to this conclusion by the fact that sore-throat, although of a mild kind, had preceded the laryngeal symptoms for some days, and still existed at the time of the patient's admission to the hospital. This accords with the ordinary history of diphtheria affecting the air-passages; it commences in the fauces, and usually, after a shorter or longer interval, creeps downwards into the larynx and trachea. The interval between the commencement of the illness and the accession of laryngeal symptoms may indeed be very brief, sometimes not exceeding a few hours, but in my experience it has more frequently been several days, though very rarely protracted beyond a week. One reason why laryngeal diphtheria appears to commence suddenly is the fact, to which I have already adverted, that the earlier symptoms of cases which ultimately becomes dangerous are often of the mildest character, and consequently sometimes altogether escape the observation of friends or attendants until the symptoms of actual croup give the alarm. Last autumn I saw, at the request of her medical attendant, a little girl, aged four years, whose indisposition had been so entirely overlooked by her parents, that they had brought her up from the country only a few hours before I saw her, and until the dyspnœa and stridulous breathing suddenly came on, and induced them to send for medical advice, were purposing to take her on to Gloucestershire. Yet not only were the tonsils enlarged, with a patch of exudation, the size of a sixpenny-piece, on each, but the fauces were generally injected, and the glands at the angles of the lower jaw swollen; showing beyond doubt that the disease must have been progressing for some days, even if we had not ascertained on inquiry that the child had had slight sore-throat for nearly a week, which had not, however, prevented her from taking her food, and going about as usual. In fact, in that case, as well as in the case we are considering, it was evident to me, when first called for an opinion, that the disease had already reached a stage in which, unless speedily relieved, the patient could not survive many hours, and in which the only possible modes of relief were either by means of the spontaneous expulsion of the false membrane, which I felt assured was choking up the larynx or trachea, or else by means of the artificial admission of air to the lungs through an opening in the trachea. Now and then, though very rarely, I have known cases of this kind recover, when apparently desperate, in consequence of the spontaneous separation and expulsion of a mass of false membrane, bearing a more or less close resemblance in shape to some portion of the air-passages. I had some time since in my possession two such portions of false membrane, one of which formed an exact cast of the lower part of the trachea and the first portion of the bronchi, and the other an equally accurate cast of the larynx and upper part of the trachea. Both had been expelled by patients who were apparently almost moribund, and in both cases the expulsion was followed by recovery. I also some years ago found in the post-mortem examination of a little girl, whom I had seen once a few hours before death, a deposit of false membrane lining the whole of the larynx, and extending nearly an inch downwards into the trachea. This false membrane formed a complete tubular cast of the parts, but was almost entirely loose, being only attached at a few points to the mucous membrane which it overlaid. Thick and dense in the larynx, it became gradually thinner in the trachea, until it terminated in an extremely thin, soft, and scarcely coherent pellicle. The child had died very suddenly a few hours after I had seen it, and the immediate cause of death appeared to have been the partial detachment of the false membrane lining the larynx, which had choked the passage, and barred the admission of air. I was much mortified to find after death that possibly the operation of tracheotomy might have saved the child's life, and that the false membrane, being loose, might not improbably have been seized with a pair of forceps, and drawn through the wound. I should add, however, that the spontaneous detachment and expulsion of the membraniform exudation, whether entire or in flakes, by no means insures the patient's recovery. The temporary relief is indeed for the most part very remarkable, and encourages the hope that the patient—who, although only a short time before apparently in the last struggle for life, may now be sleeping calmly—is on the high road to perfect convalescence; and in some instances this may really be the case. But such hopes are too often illusory, for the same tendency to repeated renewal of the false membrane by fresh exudation, which is frequently seen in diphtheritic affections of the fauces, exists also in the larynx; and, unless the local inflammation itself be upon the wane, it is too frequently found, a few hours after the expulsion of the former cast, that a new one occupies its place, and that the patient is in a worse state than before, being less able to cope with the fresh attacks of dyspnœa, and less likely to have strength to expel the obstruction. In fact, as long as the inflamed mucous membrane continues to pour out the liquid exudation which coagulates into the diphtheritic deposit, so long must the process of formation go on. It is by the persistence of this process that what was at first a mere semi transparent pellicle on the fauces becomes in the space of a few hours a dense membrane, and that the latter often increase in thickness from day to day, notwithstanding the waste going on at its free surface. And so therefore I regard it as probable, seeing the great rapidity with which the membrane re-forms upon the fauces when it has been artificially detached, that, unless the inflammatory affection be really on the decline, the processes of exudation and coagulation may go on for a time even more rapidly after the expulsion of the false membrane from the larynx than whilst it still covered the diseased surface.

To return, however, to the case of our little hospital patient. I was convinced, as already said, that the only possible chances for her life lay either in the almost immediate expulsion of the false membrane or in the speedy performance of tracheotomy. The former is, as I have explained, an event of rare occurrence, never to be counted on in any particular case, and the issue of which is exceedingly uncertain when it does happen; the latter alternative of operation has very frequently failed in such cases, and seemed especially likely to do so in a case in which the left lung was already partially consolidated, and the bronchial membrane probably inflamed. Nevertheless, considering that the child's suffering was urgent, that its death in a few hours seemed inevitable unless relief could be given by the operation, and that there were no severe constitutional symptoms to contraindicate tracheotomy, I spoke very decidedly in favor of its being performed. The event proved that in the circumstances the operation was not only justifiable, but right, for it was scarcely over when the child's breathing became comparatively tranquil, and she fell into a quiet sleep almost as soon as laid down in bed; and although it is true that in this case, as in too many others, life was not saved, it was certainly prolonged, the most urgent suffering was permanently relieved, and death came in a gentler and less distressing form than it would otherwise have done.

The immediate causes of death in this case were, doubtless, the collapse of lung and the plunging up of the trachea and primary bronchi with tenacious mucus, which the child was unable to get rid of by expectoration; for the lung-tissue, although collapsed, was not inflamed, and the bronchitis was scarcely in itself severe enough to have proved dangerous, except as a complication of the graver disease. In fact, however, I regard both the bronchitis and the collapse of lung as having resulted from the laryngeal affection; the former having probably been mainly occasioned by the gravitation downwards of the acrid fluid, from the larynx and trachea consequent upon the patient's inability to expectorate. The collapse of lung doubtless arose, as it so often does in the bronchitis of children, from the imperfect admission of air into the lungs during inspiration, partly in consequence of the obstruction in the larynx and trachea, partly from the choking up of the bronchial tubes with tenacious mucus. This latter, again, was in a great measure owing to the inability to cough it up, consequent on the want of power to take such a full inspiration as necessarily precedes the act of coughing. This was therefore eminently a case of diphtheria fatal in consequence of the local manifestations of the disease, and it was in the conviction that these constituted the real danger of the case that I entertained no doubt respecting the propriety of endeavoring to save the patient by tracheotomy.—*Lancet*, June 3, 1865.

